# Immunoinflammatory mechanisms and emerging therapies for postherpetic neuralgia

**DOI:** 10.1007/s10072-026-09354-0

**Published:** 2026-08-27

**Authors:** Shunv He, Dan Deng, Wen-fei Luo

**Affiliations:** 1 Hangzhou Linping Integrated Traditional Chinese and Western Medicine Hospital, Hangzhou, Zhejiang China; 2 Ningxia Medical University, The General Hospital of Ningxia Medical University, Yinchuan, Ningxia China; 3 804 Shengli South Street, Yinchuan, Ningxia 750004 China

**Keywords:** Postherpetic neuralgia (PHN), Herpes zoster (HZ), Immune-inflammatory mechanisms, Treatments, Neuropathic pain

## Abstract

This review examines the immunoinflammatory mechanisms underlying postherpetic neuralgia (PHN) and emerging mechanism-based therapies. PHN is increasingly recognized as a neuroimmune disorder driven by varicella-zoster virus reactivation, peripheral nerve injury, cytokine-mediated inflammation, oxidative stress, glial activation, and central sensitization. Although conventional treatments, including antidepressants, anticonvulsants, topical agents, opioids, and neuromodulation, provide symptomatic relief, they rarely modify the underlying disease process. A key scientific gap is the lack of consensus on the optimal timing, patient selection, and targets for immunomodulatory, gene-based, or RNA-based interventions to prevent the transition from acute neuroinflammation to chronic pain. Therefore, this review briefly summarizes standard-of-care therapies and focuses on emerging strategies targeting upstream mechanisms, including cytokine modulation, NLRP3 inflammasome inhibition, oxidative stress regulation, viral vector-based gene therapy, RNA interference, non-coding RNA approaches, and phenotype-guided precision medicine. We propose a stage-specific, mechanism-oriented framework for future PHN management.

## Introduction

Herpes zoster (HZ), caused by reactivation of varicella-zoster virus (VZV), predominantly affects older and immunocompromised individuals [1, 2]. Its most disabling complication, postherpetic neuralgia (PHN), occurs in approximately 10–20% of patients and manifests as persistent neuropathic pain with substantial impact on quality of life, particularly in the elderly [1, 3]. Increasing evidence indicates that PHN is not merely a residual manifestation of cutaneous infection, but a chronic neuroimmune disorder driven by persistent peripheral sensory neuronal injury and maladaptive central sensitization within the spinal cord and higher pain-processing pathways [1, 3]. Despite antiviral therapy and conventional neuropathic pain medications, clinical outcomes remain suboptimal, reflecting the limited capacity of current treatments to modify disease progression [2, 4]. Neuroinflammation—mediated by dysregulated cytokine signaling and pathological activation of immune and glial cells—has emerged as a critical mechanistic link between VZV-induced neural injury and sustained peripheral and central hyperexcitability [5].

In this review, we integrate current evidence on peripheral and central neuroimmune mechanisms in PHN, evaluate existing therapies in relation to these mechanisms, and discuss emerging immunomodulatory and gene-targeted strategies with potential disease-modifying relevance. Particular emphasis is placed on a major unresolved gap in the field: the lack of consensus regarding the optimal timing, patient selection, and mechanistic targets for immunomodulatory, gene-based, and RNA-based interventions during the transition from acute herpes zoster to chronic PHN.

## Immunoinflammatory pathogenesis of PHN

PHN arises from a complex interplay of immune and inflammatory mechanisms initiated by the reactivation of VZV. This process involves viral replication, immune dysregulation, nerve damage, and sensitization of the peripheral and central nervous systems, culminating in chronic pain.

**Fig. 1:**
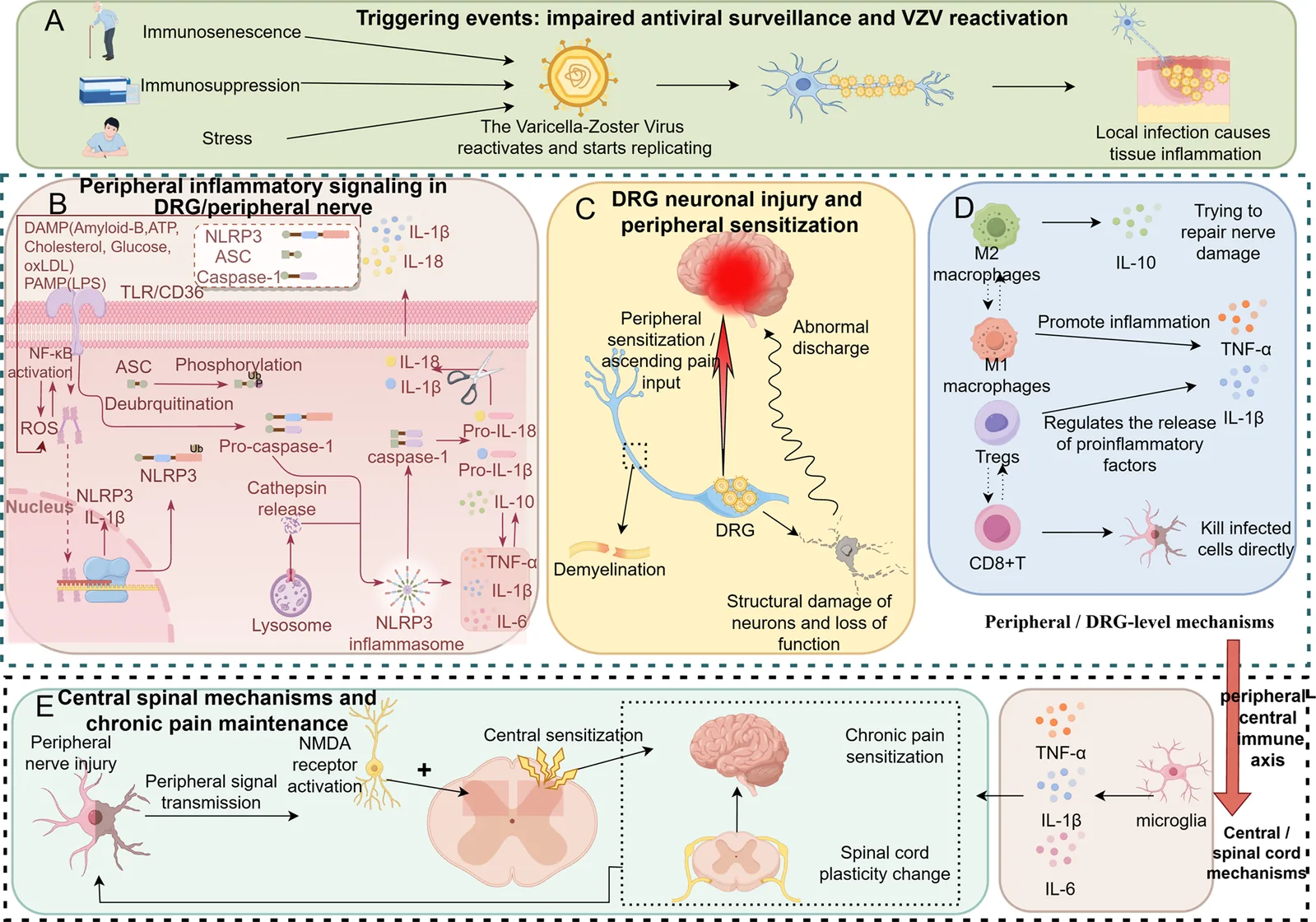
Immunoinflammatory Pathogenesis of PHN.** (A)** Immunosenescence, immunosuppression, and stress impair VZV-specific antiviral immune surveillance, leading to VZV reactivation, viral replication, axonal spread, and local tissue inflammation. **(B)** At the peripheral nerve/DRG level, VZV-induced injury and danger signals activate ROS-related inflammatory pathways and the NLRP3 inflammasome, promoting the release of IL-1β, IL-18, TNF-α, and IL-6, whereas IL-10 may exert counter-regulatory effects. **(C)** DRG neuronal injury causes demyelination, structural damage, abnormal discharges, and enhanced ascending nociceptive input, resulting in peripheral sensitization. **(D)** Peripheral immune-cell responses involve M1/M2 macrophages, Tregs, and CD8 + T cells, which regulate inflammatory injury, tissue repair, immune suppression, and antiviral cytotoxicity. **(E)** Persistent peripheral input drives central spinal mechanisms, including NMDA receptor activation, microglial cytokine release, spinal cord plasticity, and central sensitization, thereby maintaining chronic pain through a peripheral–central immune axis. By Figdraw

Figure 1 summarizes the immunoinflammatory pathogenesis of PHN and distinguishes peripheral/DRG-level mechanisms from central spinal mechanisms. The peripheral component includes impaired antiviral immune surveillance, VZV reactivation, DRG/peripheral nerve injury, inflammasome activation, cytokine release, macrophage and T-cell responses, and peripheral sensitization. The central component highlights persistent nociceptive input, NMDA receptor activation, microglial cytokine release, spinal cord plasticity, and central sensitization. Together, these processes illustrate a peripheral–central immune axis that links VZV-induced peripheral neuroinflammation to chronic pain maintenance.

### Viral activation and nerve damage

VZV, the virus responsible for HZ and its complication PHN, remains dormant in peripheral sensory ganglia after primary infection. Upon reactivation—commonly triggered by aging, immunosuppression, or stress—VZV replicates in sensory neurons, spreads along axons to innervated tissues, and induces marked inflammation and neuronal injury [6, 7]. Importantly, neuronal damage in PHN is not solely due to direct viral cytopathic effects but is largely driven by an exaggerated and dysregulated host immune response. Macrophages and T cells infiltrate the dorsal root ganglia (DRG), releasing pro-inflammatory cytokines that enhance neuronal excitability and impair axonal integrity, thereby amplifying pain signaling [6, 7]. Whether persistent pain results from ongoing low-level viral activity or from sustained neuroimmune dysregulation after viral clearance remains an unresolved but clinically relevant question.

VZV replication within the DRG activates neuroinflammatory cascades and upregulates neuronal injury markers, including ATF-3, Nav1.8, and neuropeptide Y (NPY) [8, 9]. These molecular alterations lower activation thresholds, promote ectopic discharges, and sustain peripheral sensitization. Such changes resemble mechanisms observed in other neuropathic pain states, yet the virus-driven immune activation in PHN introduces a distinct pathogenic dimension.

Central sensitization further contributes to the chronicity of PHN. Persistent nociceptive input enhances dorsal horn neuron activity and induces long-term synaptic plasticity changes in the central nervous system [9, 10]. Once established, these central alterations may become partially independent of peripheral input, potentially explaining why antiviral therapy during the acute phase does not consistently prevent PHN progression.

Thus, viral reactivation, immune-mediated inflammation, and maladaptive neuronal sensitization form an interconnected pathological process [11, 12]. Current therapies mainly target symptomatic relief rather than interrupting this cascade, highlighting the need for early interventions that modulate neuroinflammation and prevent irreversible central plasticity.

### The role of inflammatory factors in PHN

TNF-α plays a central role in the pathogenesis of PHN by bridging peripheral nerve injury and sustained neuroinflammation. It promotes myelin degradation and axonal injury, thereby facilitating neuropathic pain development. In the DRG, TNF-α enhances nociceptor excitability by increasing intracellular calcium influx and upregulating voltage-gated sodium channels, which lowers activation thresholds and promotes ectopic firing [13]. Elevated TNF-α levels in cerebrospinal fluid (CSF) and DRG tissues of PHN patients support its contribution to peripheral sensitization [13]. Experimental models further demonstrate that TNF-α induces hyperalgesia and allodynia through amplification of synaptic transmission and inflammatory signaling.

Clinically, systemic TNF-α levels during the acute phase of HZ appear to correlate with PHN risk, as patients with lower early TNF-α expression are less likely to develop chronic neuralgia [14, 15]. Observational data suggesting reduced PHN incidence in patients receiving TNF-α inhibitors provide indirect evidence of its pathogenic relevance [16]. However, this also raises a therapeutic dilemma: while TNF-α blockade may attenuate neuroinflammation, excessive immunosuppression could increase susceptibility to viral reactivation. Thus, the timing and context of TNF-α modulation remain critical and warrant further prospective investigation. Moreover, the interaction between TNF-α and neurotrophic mediators such as nerve growth factor (NGF) suggests that TNF-α may not only initiate inflammation but also sustain maladaptive neuronal plasticity [13], reinforcing its potential as a disease-modifying target rather than merely a biomarker.

The interleukin (IL) family further shapes the inflammatory milieu in PHN by balancing pro- and anti-inflammatory signaling. IL-1β and IL-6 are key drivers of neuroinflammation, enhancing nociceptor sensitization and glial activation within the DRG and spinal cord. Elevated IL-6 levels during acute HZ are strongly associated with hyperalgesia and increased risk of PHN development [15, 17], suggesting that IL-6 may serve as a predictive biomarker for chronicity. IL-1β amplifies inflammatory cascades and promotes persistent neuronal hyperexcitability [13], potentially contributing to the transition from acute nociceptive pain to chronic neuropathic pain.

In contrast, IL-10 is generally considered an anti-inflammatory cytokine that may suppress pro-inflammatory cytokine production and contribute to immune resolution and neural recovery [14, 18]. However, elevated IL-10 levels have also been observed in patients with severe HZ manifestations or prolonged neuralgia, suggesting that IL-10 may reflect compensatory anti-inflammatory activation or persistent immune dysregulation rather than simple protection [18]. Therefore, IL-10 should be interpreted as a stage- and context-dependent marker of immune regulation, rather than as a uniformly protective therapeutic target. Meanwhile, IL-18 has emerged as a potential genetic and inflammatory risk factor, linking innate immune activation with sustained neuroinflammation [19]. Although less extensively studied, its association with pain chronicity suggests that inflammasome-related pathways may represent upstream therapeutic targets.

Inflammatory factors play a critical role in the pathogenesis of PHN, contributing to pain persistence and central sensitization. Table 1 lists the key inflammatory cytokines, including TNF-α, IL-6, and IL-10, along with their sources, mechanisms of action, and potential therapeutic targets.

**Table 1:** Inflammatory factors associated with PHN

Inflammatory factor	Source	Mechanism of action	Potential therapeutic targets	References
TNF-α	Host immune cells (e.g., macrophages)	Induces myelin degradation and axonal injury; promotes calcium influx into neurons, increasing nociceptor excitability; upregulates sodium channel expression, amplifying synaptic activity.	TNF-α inhibitors (e.g., adalimumab); reducing TNF-α-mediated inflammation.	[14]
IL-6	Macrophages, T cells	Activates nociceptors and glial cells in DRG, enhancing central sensitization and peripheral inflammation; elevated IL-6 during acute HZ is strongly associated with PHN risk.	IL-6 blockers; modulation of its receptor signaling pathways.	[17]
IL-1β	Monocytes/Macrophages	Enhances nociceptor excitability and promotes the release of other inflammatory mediators, contributing to chronic pain persistence.	NLRP3 inflammasome inhibitors; inhibition of IL-1β production.	[13]
IL-10	Anti-inflammatory macrophages	Suppresses pro-inflammatory cytokines and may contribute to immune resolution; elevated IL-10 may also indicate compensatory anti-inflammatory activation or persistent immune dysregulation.	IL-10 activators or gene therapy.	[18]
IL-18	Monocytes, macrophages	Contributes to neuroinflammation and central sensitization, increasing genetic risk of PHN; associated with chronic pain persistence.	IL-18 inhibitors; modulation of its signaling pathway.	[19]

Abbreviations: *TNF-α* Tumor Necrosis Factor-Alpha, *IL-6* Interleukin-6, *IL-1β* Interleukin-1 Beta, *IL-10* Interleukin-10, *IL-18* Interleukin-18, *DRG* Dorsal Root Ganglia, *HZ* Herpes Zoster, *PHN* Postherpetic Neuralgia, *NLRP3* NOD-, LRR-, and Pyrin Domain-Containing Protein 3

Collectively, inflammatory cytokines in PHN do not act independently but form a dynamic network that influences the transition from acute viral inflammation to persistent central sensitization. From a clinical perspective, defining cytokine profiles during the early stage of HZ may help stratify patients at high risk for PHN and guide targeted immunomodulatory interventions. Future research should therefore move beyond descriptive cytokine measurement toward mechanism-driven trials that clarify optimal timing, dosing, and patient selection for cytokine-targeted therapies.

### The role of immune cells in PHN

Immune cells critically shape the transition from acute VZV infection to persistent neuropathic pain by orchestrating both peripheral and central neuroinflammatory responses. In PHN, macrophages, T cells, and microglia form an interconnected cellular network that sustains neuronal hyperexcitability and maladaptive plasticity.

Macrophages exhibit functional heterogeneity during PHN progression. Pro-inflammatory M1 macrophages infiltrate the DRG during the acute phase and release cytokines such as TNF-α and IL-1β, which enhance nociceptor excitability and promote hyperalgesia and allodynia [20, 21]. These mediators also disrupt axonal integrity and facilitate ectopic discharges. Conversely, M2 macrophages secrete IL-10 and contribute to tissue repair and resolution of inflammation [22].However, in many PHN patients, the balance appears skewed toward a sustained M1-dominant phenotype, suggesting that insufficient immune resolution rather than excessive initial inflammation may underlie chronicity. Whether therapeutic strategies that promote macrophage polarization toward an M2 phenotype can prevent PHN remains an open and clinically relevant question.

T cells further modulate this peripheral immune environment. CD8 + T cells target VZV-infected neurons, contributing to viral control but also potentially exacerbating neuronal injury [5, 23]. Persistent T cell infiltration within the DRG may sustain low-grade inflammation even after rash resolution. Regulatory T cells (Tregs) increase during acute HZ and PHN [24], reflecting an attempt to restrain inflammation; however, excessive Treg activity may dampen antiviral immunity and delay viral clearance. This duality highlights a central paradox in PHN immunopathogenesis: insufficient viral control and excessive immune-mediated injury may coexist. Clinically, this may partly explain why both immunosuppressed and hyperinflammatory states can predispose patients to prolonged neuralgia.

At the central level, microglia act as key amplifiers of pain signaling. Following persistent peripheral input, activated microglia release TNF-α, IL-1β, and other mediators that enhance synaptic transmission and facilitate central sensitization [25, 26]. Microglial activation is often accompanied by astrocytic responses, together promoting spinal cord plasticity changes that maintain chronic pain independent of ongoing peripheral injury. Importantly, experimental inhibition of microglial activation alleviates neuropathic behaviors in animal models [25, 26], suggesting that central immune modulation may be particularly relevant in established PHN, when peripheral antiviral strategies are less effective.

Collectively, macrophages and T cells initiate and shape peripheral neuroinflammation, whereas microglia and astrocytes consolidate central sensitization. Rather than acting independently, these immune cells form a dynamic peripheral–central immune axis that determines whether acute viral inflammation resolves or progresses to chronic neuropathic pain. Identifying stage-specific immune signatures may therefore enable more precise immunomodulatory interventions in PHN management.

### Oxidative stress and nerve damage

Oxidative stress represents a critical interface between viral-induced inflammation and persistent neuronal dysfunction in PHN. Excessive production of reactive oxygen species (ROS) during acute neuroinflammation damages lipids, proteins, and mitochondrial DNA, thereby compromising neuronal integrity. ROS can directly activate nociceptive neurons via TRPA1 channels, lowering pain thresholds and enhancing peripheral sensitization [27]. Mitochondrial dysfunction induced by oxidative stress further impairs ATP production and axonal transport, limiting neuronal repair capacity and potentially predisposing injured neurons to chronic hyperexcitability. Clinically, reduced systemic antioxidant markers such as uric acid and albumin in PHN patients suggest a sustained imbalance between oxidative injury and endogenous protective mechanisms [28], supporting the relevance of oxidative stress beyond experimental models.

Importantly, oxidative stress does not act in isolation but amplifies immune signaling pathways. Recent evidence from broader neuroinflammatory and neurodegenerative contexts further supports the close interaction between oxidative stress, microglial activation, and chronic inflammatory signaling. Carotenoids have been summarized as natural antioxidant and anti-inflammatory compounds capable of modulating oxidative stress and inflammation-related pathways in neurological disorders, providing mechanistic support for exploring redox-modulating strategies in PHN-related neuroinflammation [29]. Similarly, sesamol, a natural phenolic lignan, has shown neuroprotective potential through regulation of oxidative stress and neuroinflammation, although its direct therapeutic value in PHN remains to be validated [30]. ROS are potent activators of the NLRP3 inflammasome in immune and glial cells, leading to the release of IL-1β and IL-18 and reinforcing neuroinflammatory cascades [31]. This ROS–NLRP3 axis provides a mechanistic bridge between metabolic stress and cytokine-driven sensitization. Furthermore, ROS-induced activation of inducible nitric oxide synthase (iNOS) enhances nitrosative stress, creating a feed-forward cycle that sustains both peripheral and central inflammation [32]. Such interactions suggest that oxidative stress may function as an upstream driver of immune dysregulation rather than merely a downstream byproduct.

Oxidative stress also interferes with neuronal autophagy, disrupting cellular homeostasis and promoting degeneration. Although increased autophagic activity has been observed in PHN models, this response may reflect maladaptive stress rather than effective repair [32]. From a therapeutic perspective, antioxidants and mitochondrial-targeted interventions have demonstrated analgesic effects in preclinical studies; however, their clinical translation in PHN remains limited. Mitoquinone (MitoQ), a mitochondria-targeted antioxidant, has been shown to attenuate pain hypersensitivity, glial activation, oxidative stress, and pro-inflammatory cytokine expression in a vincristine-induced neuropathic pain model, indicating its potential relevance to neuropathic pain mechanisms rather than PHN specifically [33]. In humans, MitoQ has progressed to clinical studies in non-PHN conditions, including chronic hepatitis C-related liver injury and age-related vascular dysfunction [34, 35]. Nevertheless, to our knowledge, no PHN-specific clinical trial has yet evaluated MitoQ or similar mitochondria-targeted antioxidants. This is consistent with recent reviews of investigational PHN drugs, in which advanced clinical candidates primarily target ion channels, AT2R, AAK1, LANCL, NMDA receptors, opioidergic signaling, or NGF, rather than mitochondrial ROS directly [36]. Therefore, although mitochondrial ROS represents a mechanistically attractive upstream target linking oxidative stress, NLRP3 inflammasome activation, and impaired autophagy, the therapeutic relevance of MitoQ in PHN remains preclinical and requires disease-specific translational validation.

Overall, oxidative stress integrates metabolic dysfunction with immune activation and synaptic plasticity changes in PHN. Targeting this pathway may therefore require combined modulation of redox balance and inflammatory signaling rather than isolated antioxidant therapy.

## Current standard-of-care management and its limitations

Current PHN management remains largely symptom-oriented, and established pharmacological options are broadly consistent with neuropathic pain treatment recommendations, although their evidence strength and clinical positioning differ across drug classes [37, 38]. Existing pharmacological options include antidepressants, gabapentin or pregabalin, topical lidocaine, high-concentration capsaicin patches, tramadol, and selected opioids, which primarily suppress neuronal hyperexcitability, enhance descending inhibitory control, or reduce peripheral nociceptive input [4, 39–44]. However, these therapies mainly target pain transmission and do not directly reverse VZV-induced neuronal injury, cytokine-mediated inflammation, oxidative stress, glial activation, or maladaptive central sensitization, which are key mechanisms implicated in PHN persistence [3, 5, 9, 13, 25, 26, 45].

Non-pharmacological strategies, including transcutaneous electrical nerve stimulation (TENS), spinal cord stimulation, acupuncture, moxibustion, and psychological interventions, provide additional options for selected patients. These approaches may modulate peripheral input, spinal pain processing, central sensitization, or affective pain amplification, and may be particularly useful when systemic pharmacotherapy is limited by adverse effects, older age, comorbidity, or incomplete analgesic response [46–51]. Nevertheless, current evidence supports these interventions primarily as symptomatic or adjunctive strategies, and their ability to modify the long-term neuroimmune trajectory of PHN remains unproven, especially after central sensitization has become established [3, 9, 45].

The role of psychological interventions is particularly relevant within a biopsychosocial framework. Stress is a recognized trigger for both VZV reactivation and pain exacerbation [52]. Mechanistically, chronic psychological stress may activate the hypothalamic–pituitary–adrenal axis and sympathetic nervous system, leading to altered glucocorticoid and catecholamine signaling, impaired cell-mediated antiviral immunity, and reduced VZV-specific immune surveillance [6, 7, 52]. This process may facilitate VZV reactivation in sensory ganglia and amplify the immunoinflammatory cascade described above, including DRG inflammation, macrophage and T-cell infiltration, pro-inflammatory cytokine release, and subsequent glial activation [5, 13, 20, 21, 23, 25, 26]. In established PHN, stress-related neuroendocrine dysregulation may further enhance central sensitization, sleep disturbance, and affective pain processing [45, 52–54]. Thus, CBT, MBSR, and related psychological interventions should not be viewed merely as coping strategies, but as adjunctive approaches that may help reduce neuroendocrine and affective amplification of PHN-related pain [50, 51].

**Fig. 2:**
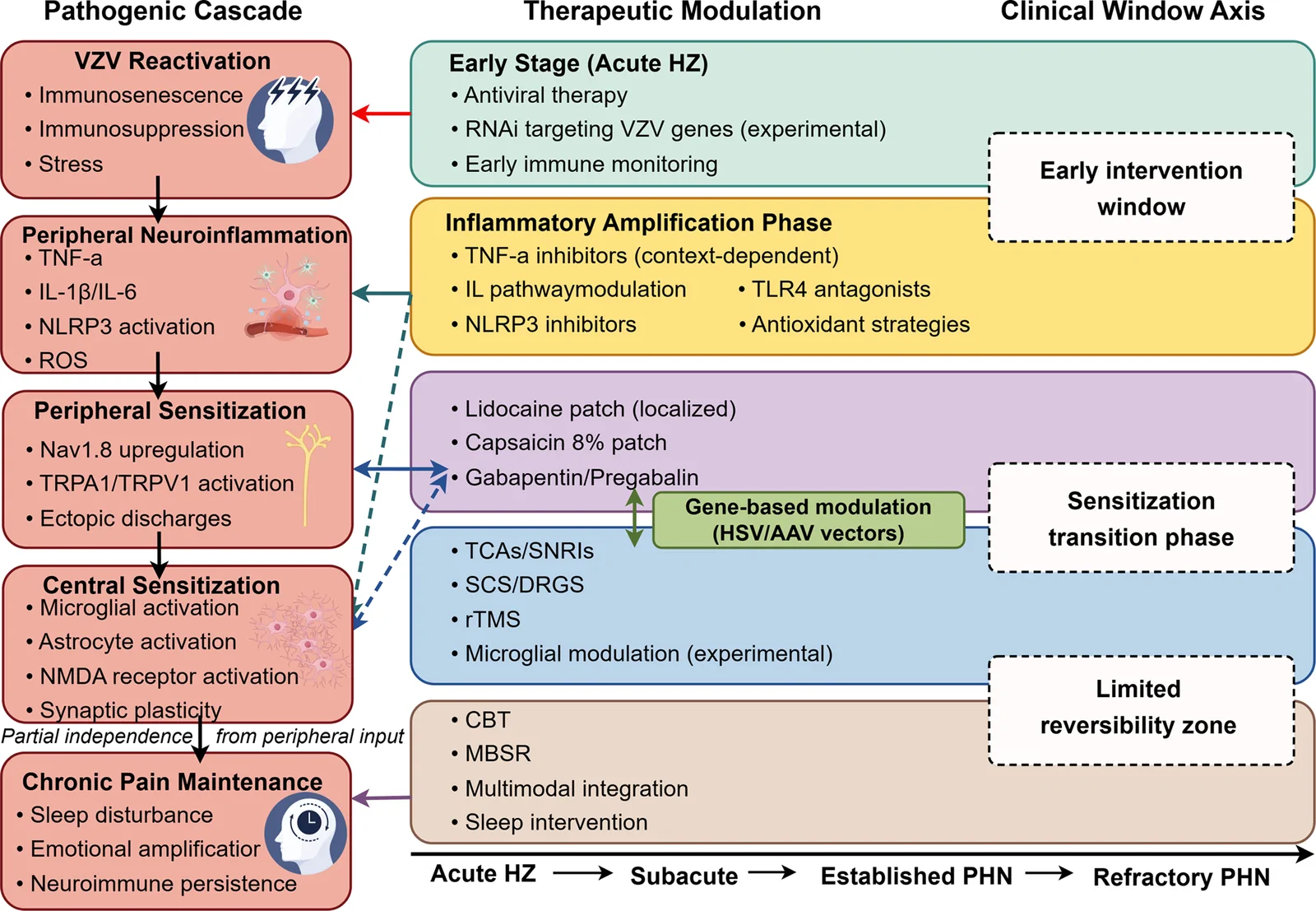
Stage-specific therapeutic modulation in PHN. The left panel illustrates the sequential pathogenic cascade from VZV reactivation to chronic pain maintenance. The middle panel depicts mechanism-oriented therapeutic strategies targeting different stages of disease progression. Early antiviral and immunomodulatory interventions may attenuate inflammatory amplification, while peripheral-targeted therapies suppress ectopic discharges. Once central sensitization is established, neuromodulatory strategies partially modulate spinal plasticity, although reversibility becomes limited. Psychological and multimodal approaches address chronic pain maintenance and functional impairment. This framework emphasizes stage-dependent and mechanism-informed therapeutic integration rather than isolated symptomatic control. By Figdraw

Therefore, standard therapies should be viewed as essential components of PHN symptom control, but not as sufficient solutions for the immunoinflammatory and neuroplastic mechanisms that drive pain persistence. Detailed mechanisms, clinical roles, and limitations of standard-of-care treatments are summarized in Table 2. To integrate these therapeutic limitations with disease progression, Fig. 2 provides a stage-specific framework linking the pathogenic cascade of PHN with mechanism-oriented interventions. This framework highlights why conventional analgesic strategies remain clinically necessary but insufficient, and why emerging immunomodulatory, molecular, and phenotype-guided approaches may be needed to intervene earlier in the transition from acute VZV-induced neuroinflammation to chronic pain maintenance. These considerations provide the rationale for the following discussion of emerging mechanism-based therapies.

**Table 2:** Standard-of-care therapies for PHN

Treatment	Main mechanism	Clinical role	Major limitations	References
TCAs/SNRIs	Enhance descending inhibitory pathways; modulate norepinephrine and serotonin signaling	First-line systemic treatment for neuropathic pain	Anticholinergic effects, sedation, cardiovascular risk, limited tolerability in elderly patients	[4, 37–39]
Gabapentin/Pregabalin	Bind α2δ subunits of voltage-gated calcium channels and reduce excitatory neurotransmitter release	First-line treatment for established PHN	Dizziness, somnolence, edema, dose titration issues, incomplete pain relief	[4, 37–40]
5% Lidocaine patch	Blocks peripheral sodium channels and reduces ectopic discharges	Localized allodynia; useful in elderly or medically complex patients	Partial efficacy; local skin reactions	[4, 37, 38, 41]
8% Capsaicin patch	TRPV1-mediated nociceptor defunctionalization	Localized peripheral neuropathic pain	Burning, erythema, limited effect on central sensitization	[4, 37, 38, 44]
Tramadol/Opioids	µ-opioid receptor activation; tramadol also inhibits monoamine reuptake	Rescue or second-line therapy for refractory pain	Sedation, constipation, tolerance, dependency risk	[37, 38, 42, 43]
TENS	Modulates peripheral input and spinal pain processing	Non-invasive adjunctive therapy	Variable response; optimal parameters remain unclear	[46]
SCS/DRGS	Neuromodulation of spinal or dorsal root ganglion pain circuits	Refractory PHN with central sensitization features	Invasive, costly, potential implantation-related complications	[47, 59]
Acupuncture/Moxibustion	Modulates peripheral and central pain pathways; possible inflammatory regulation	Integrative adjunctive treatment	Heterogeneous protocols and variable evidence quality	[48, 49]
CBT/MBSR	Reduces pain catastrophizing, mood disturbance, and affective pain amplification	Patients with anxiety, depression, sleep disturbance, or high pain-related distress	Limited large-scale PHN-specific trials	[50–53]

## Emerging mechanism-based therapies and translational directions

Unlike current standard-of-care therapies, which mainly provide symptomatic analgesia, emerging mechanism-based strategies aim to intervene more directly in the upstream immunoinflammatory, redox-related, glial, and molecular processes that sustain PHN. These approaches include cytokine-targeted immunomodulation, inflammasome inhibition, oxidative stress and mitochondrial modulation, viral vector-based gene therapy, RNA interference (RNAi), non-coding RNA-targeted therapy, and phenotype-guided precision medicine. Importantly, most of these strategies remain exploratory, preclinical, or supported by indirect evidence from non-PHN neuropathic pain models; therefore, their translational maturity should be clearly distinguished from established clinical care. Table 3 summarizes the target mechanisms, representative strategies, stages of development, and major limitations of experimental and pipeline therapies targeting PHN-related mechanisms.

**Table 3:** Experimental and pipeline therapies targeting PHN-related mechanisms

Experimental/pipeline therapy	Target mechanism	Representative strategy	Stage of development	Key limitations	References
Cytokine-targeted immunomodulation	TNF-α, IL-6, IL-1β-mediated neuroinflammation	TNF-α inhibitors, IL pathway modulation	Observational/indirect clinical evidence; not PHN-approved	Risk of impaired antiviral immunity; unclear timing and patient selection	[13–19, 55–58]
NLRP3 inflammasome inhibition	ROS–NLRP3–IL-1β/IL-18 inflammatory cascade	NLRP3 inhibitors; inflammasome-modulating approaches	Preclinical/exploratory clinical evidence	Lack of PHN-specific randomized trials	[19, 31, 32]
Oxidative stress and mitochondrial modulation	ROS, mitochondrial dysfunction, glial activation	MitoQ, antioxidants, redox-modulating compounds	Mostly preclinical or indirect evidence	PHN-specific efficacy and safety unvalidated	[27, 28], 33– [36]
Natural antioxidant/anti-inflammatory compounds	Oxidative stress, microglial activation, inflammatory signaling	Carotenoids, sesamol, nanoformulations	Exploratory/preclinical or non-PHN evidence	Limited disease-specific validation	[29, 30]
Viral vector-based gene therapy	Long-term modulation of sensory neuron excitability and neuroinflammation	HSV-PENK, AAV-GAD65/GDNF/IL-10, TNF-soluble receptor vectors	PHN-like preclinical models; related vectors in Phase I or early clinical trials for non-PHN pain	Vector safety, immune response, dose control, long-term expression	[60–65]
RNA interference	Viral replication, ion channel expression, inflammatory mediators	ORF7-siRNA, SCN9A/Nav1.7 RNAi, TNF-α shRNA	In vitro/preclinical; PHN-specific clinical validation lacking	Delivery barriers, off-target effects, durability	[66–69]
Non-coding RNA-targeted therapy	lncRNA/miRNA regulation of astrocyte activation and neuronal excitability	KCNA2-AS/STAT3 targeting; miRNA-based approaches	Preclinical PHN-related models	Early-stage evidence; translational feasibility unclear	[70, 71]
Phenotype-guided precision medicine	Patient stratification based on inflammatory profile, sensory phenotype, and disease stage	Cytokine profiling, pain mapping, quantitative sensory testing	Conceptual/translational framework	Requires prospective validation	[13– [19, 41, 44, 45, 47], 50– [54, 59], 66– [75]

### Targeted immunoinflammatory therapies

Given the central role of cytokine signaling, immune-cell infiltration, and glial activation in PHN pathogenesis, targeted immunoinflammatory therapy represents a logical extension from mechanistic understanding to potential disease-modifying intervention [9]. However, immunomodulation in PHN should not be viewed as simple anti-inflammatory escalation. Because PHN arises from VZV-triggered neural inflammation, effective intervention must suppress pathological neuroinflammation without compromising VZV-specific antiviral immune surveillance. Therefore, the key unresolved question is not only whether inflammation should be inhibited, but when, in whom, and which immune axis should be modulated.

TNF-α illustrates this therapeutic dilemma. Mechanistically, TNF-α contributes to peripheral sensitization by enhancing nociceptor excitability, inflammatory amplification, and maladaptive neuronal plasticity [13]. Clinical evidence also suggests its relevance: altered TNF-α responses during acute HZ have been associated with PHN risk, and patients receiving anti-TNF-α therapy for autoimmune diseases may show a lower incidence of PHN [14–16]. Nevertheless, TNF-α blockade may impair antiviral immune surveillance and increase the risk of herpes zoster reactivation [55]. Thus, TNF-α inhibition is unlikely to be suitable as a broad PHN therapy; if considered, it would require careful patient selection, exclusion of uncontrolled viral replication or severe immunosuppression, and possibly integration with antiviral treatment during the acute-to-subacute transition.

The timing of immunoinflammatory intervention is critical. Antiviral therapy is prioritized during the acute eruptive phase, ideally within 72 h after rash onset [56]. However, antiviral therapy alone does not reliably prevent PHN, and corticosteroids have not shown consistent benefit in preventing PHN progression [57, 58]. These findings suggest that viral suppression may be insufficient once DRG inflammation, cytokine release, and nociceptive sensitization have been initiated. A plausible but unvalidated therapeutic window may exist during the subacute phase, when viral replication is better controlled but neuroinflammatory amplification may still be modifiable [9, 45]. Once established PHN develops, pain may become less dependent on ongoing viral activity and more dependent on central sensitization and neuroplastic maintenance, making symptomatic treatment and selected neuromodulatory approaches more clinically relevant [45, 56, 59].

Interleukin-targeted strategies are also complex because cytokines may represent both pathogenic mediators and compensatory immune responses. IL-1β and IL-6 are generally associated with pro-inflammatory sensitization, nociceptor hyperexcitability, and glial activation, whereas IL-10 may contribute to immune resolution and neural recovery [13–18]. However, elevated IL-10 has also been linked to severe HZ manifestations or prolonged neuralgia, suggesting that it may reflect persistent immune dysregulation rather than simple protection [14, 18]. IL-18 further links innate immune activation with PHN susceptibility [19]. These findings argue against treating individual cytokines as isolated targets and instead support dynamic cytokine profiling according to disease stage, viral control status, and pain phenotype.

### Inflammasome, oxidative stress, and glial modulation

Inflammasome activation, oxidative stress, and glial responses constitute an interconnected therapeutic axis in PHN. VZV-induced neuronal injury and immune activation can increase ROS, which may damage neuronal structures, impair mitochondrial function, and enhance nociceptive sensitivity [27, 28]. More importantly, ROS may promote NLRP3 inflammasome activation, resulting in IL-1β and IL-18 release and further neuroinflammatory amplification [31]. Therefore, the ROS–NLRP3–glial axis provides a mechanistic bridge between metabolic stress, innate immune activation, and chronic pain maintenance.

From a therapeutic perspective, NLRP3 inflammasome inhibition is attractive because it may target upstream inflammatory amplification rather than downstream pain transmission. However, current evidence remains exploratory. Clinical observations involving repetitive transcranial magnetic stimulation combined with acupuncture have reported changes in NLRP3-related markers in neuropathic pain [31], but these findings do not establish NLRP3 inhibition as a validated PHN therapy. Future studies should clarify whether inflammasome modulation directly alters PHN-related neuroimmune mechanisms or only accompanies symptomatic improvement.

Oxidative stress and mitochondrial dysfunction are similarly promising but insufficiently validated therapeutic targets. MitoQ has shown analgesic and anti-inflammatory effects in a vincristine-induced neuropathic pain model by reducing oxidative stress, mitochondrial dysfunction, glial activation, and pro-inflammatory cytokine expression [33]. In humans, MitoQ has been evaluated in non-PHN conditions such as chronic hepatitis C-related liver injury and age-related vascular dysfunction [34, 35]. However, no PHN-specific clinical trial has yet confirmed whether mitochondrial antioxidant therapy can prevent PHN, reverse established pain, or modify disease progression. Thus, mitochondrial ROS modulation should currently be regarded as a mechanistically plausible pipeline strategy rather than an evidence-based PHN treatment.

Natural antioxidant and anti-inflammatory compounds provide additional exploratory directions. Carotenoids and sesamol-based nanoformulations have been discussed as antioxidant, anti-inflammatory, and neuroprotective strategies in broader neurological contexts [29, 30]. Nevertheless, their relevance to PHN remains indirect, and future studies must determine whether these agents can reach sensory ganglia or spinal targets, modulate redox–immune signaling at meaningful concentrations, and demonstrate efficacy in PHN-specific models. Overall, therapies targeting the ROS–NLRP3–glial axis are promising because they address upstream immune-metabolic mechanisms, but their clinical value will depend on PHN-specific validation of efficacy, safety, dosing, and therapeutic timing.

### Viral vector-based gene therapy

Viral vector-based gene therapy represents one of the most mechanism-oriented emerging strategies for PHN because it may provide localized and long-lasting modulation of sensory neuronal excitability and neuroimmune signaling. Sensory ganglia are particularly relevant targets, as VZV reactivation, neuronal injury, ectopic discharge generation, and early immune-cell infiltration converge within the dorsal root ganglia. Unlike conventional analgesics, which mainly suppress pain transmission, viral vector-based approaches may deliver analgesic, inhibitory, neuroprotective, or anti-inflammatory transgenes directly to pain-relevant neural structures.

HSV vectors are especially attractive because of their neurotropism and ability to transduce sensory neurons. In a VZV-induced rat model of PHN-like pain, HSV-mediated delivery of the human preproenkephalin gene reduced mechanical allodynia and thermal hyperalgesia, supporting enkephalin as a relevant analgesic transgene for PHN-oriented gene therapy [60]. This evidence is important because it derives from a VZV-related pain model rather than a generic neuropathic pain model. However, it remains preclinical and does not establish clinical efficacy in human PHN.

AAV-based strategies may offer complementary advantages, including relatively stable transgene expression and the possibility of combination gene delivery. Experimental chronic pain studies have explored glutamic acid decarboxylase isoforms, especially GAD65/GAD67, to enhance inhibitory GABAergic signaling, as well as IL-10, GDNF, and TNF-soluble receptor strategies to suppress neuroimmune activation or promote neuronal repair [61–63]. These transgenes are mechanistically relevant to PHN because they correspond to key pathological processes discussed above, including neuronal hyperexcitability, insufficient inhibitory control, inflammatory amplification, and impaired neural recovery.

Despite this rationale, clinical translation remains limited and largely non-PHN-specific. The HSV-based enkephalin vector NP2 has been evaluated in a phase I study for intractable cancer pain rather than PHN [64]. Similarly, KLS-2031, an AAV-based combination gene therapy strategy, has been evaluated in a Phase 1/2a trial for neuropathic pain from lumbosacral radiculopathy rather than PHN [65]. The ClinicalTrials.gov record identifies the condition as neuropathic pain from lumbosacral radiculopathy and the intervention as KLS-2031, with an actual enrollment of 18 participants. Therefore, no viral vector-based gene therapy has yet been clinically validated specifically for PHN. Major barriers include vector safety, immune responses, dose control, long-term expression stability, target specificity, and the possibility that established central sensitization may limit reversibility if gene therapy is applied too late. Future studies should prioritize PHN-specific models, stage-specific delivery windows, sensory ganglion-targeted administration, and long-term safety monitoring.

### RNAi and non-coding RNA-targeted therapy

RNAi and non-coding RNA-targeted strategies offer a more target-specific approach for PHN-related mechanisms than conventional analgesics or broad immunomodulation. In principle, RNA-based therapies may act at three levels: inhibiting VZV replication during the early HZ stage, reducing neuronal hyperexcitability by targeting ion channel expression, and modulating inflammatory or glial signaling involved in chronic pain maintenance. However, their therapeutic relevance depends strongly on disease stage, molecular target, and delivery efficiency.

In the antiviral context, RNAi is most relevant before PHN becomes established. Recombinant ORF7-siRNA delivered by flexible nano-liposomes inhibited VZV infection and reduced viral copy numbers in vitro and in a three-dimensional human epidermal skin model [66]. This supports the feasibility of RNAi-based antiviral intervention, but its likely window is early HZ rather than established PHN, where pain may be driven more by neuronal injury, neuroinflammation, and central sensitization than by active viral replication.

Beyond viral targets, RNA-based strategies may regulate pain-related neuronal and inflammatory pathways. SCN9A/Nav1.7 is relevant because Nav1.7 contributes to nociceptor excitability; experimental evidence shows that modulation of Nav1.7-related pathways can reduce pain hypersensitivity in non-PHN pain models [67, 68]. Similarly, lentiviral shRNA-mediated silencing of TNF-α in the dorsal root ganglion reduced mechanical allodynia, inflammatory gene expression, and neuronal loss in a neuropathic pain model [69]. These findings are mechanistically relevant to PHN, but they remain largely extrapolated from non-PHN models and require disease-specific validation.

Non-coding RNA pathways provide another emerging direction. In PHN-related models, targeting the lncRNA KCNA2-AS/STAT3 axis reduced astrocyte activation and neuropathic behaviors, linking RNA regulation to glial activation and central sensitization [70]. Distinct serum miRNA profiles between acute HZ and PHN patients further suggest that RNA signatures may support patient stratification or biomarker-guided therapeutic design [71]. Overall, RNA interference and non-coding RNA-targeted therapy remain experimental but highly mechanism-oriented. Future studies should clarify delivery to sensory ganglia or spinal targets, off-target effects, immune activation, durability, therapeutic timing, and whether RNA-based interventions can modify PHN-specific neuroimmune mechanisms rather than only reduce pain behavior.

### Precision medicine and stage-specific treatment selection

Given the mechanistic heterogeneity of PHN, future management should move beyond uniform analgesic escalation toward stage-specific and phenotype-guided treatment selection. A practical precision-medicine framework should integrate disease stage, cytokine profile, sensory phenotype, pain distribution, psychosocial burden, and prior treatment response to estimate whether an individual patient is predominantly driven by active neuroinflammation, peripheral nociceptor hyperexcitability, established central sensitization, or affective pain amplification.

In the acute and subacute HZ stages, the main goal is to prevent transition to chronic PHN. Cytokine profiles may help identify high-risk patients, particularly when elevated TNF-α, IL-1β, IL-6, or IL-18 suggests ongoing pro-inflammatory sensitization, whereas IL-10 may reflect compensatory anti-inflammatory regulation or persistent immune dysregulation [13–19]. However, these biomarkers should currently be regarded as adjunctive risk-stratification tools rather than validated treatment algorithms. Their value depends on whether longitudinal immune profiling can distinguish patients likely to recover from those who may benefit from early immunomodulatory, antiviral-adjacent, or RNA-based interventions.

In established PHN, treatment selection should be matched to the dominant pain mechanism. Patients with localized dermatomal pain, prominent mechanical allodynia, and preserved correspondence with the original rash may be more suitable for peripheral-targeted strategies, particularly topical lidocaine and high-concentration capsaicin patches [41, 44, 72–75]. In contrast, patients with refractory pain, widespread allodynia or hyperalgesia, poor response to peripheral blockade, sleep disturbance, emotional amplification, or features of central sensitization may require centrally directed or multimodal approaches, including neuromodulation and psychological interventions [45, 47, 50–54, 59]. Thus, a capsaicin patch and SCS should not be viewed simply as sequential options after treatment failure; rather, they represent different mechanism-matched strategies, with capsaicin targeting peripheral nociceptor-driven pain and SCS targeting centrally maintained or refractory pain states.

Emerging molecular therapies should also be positioned within this stage-specific framework. Antiviral RNAi is likely to be most relevant during active viral replication, whereas neuronal excitability-, inflammatory-, or glial-targeted RNA strategies may be more relevant after peripheral injury and central sensitization emerge [66–71]. Similarly, immunomodulatory, inflammasome-targeted, antioxidant, gene-based, and RNA-based interventions should not be evaluated as interchangeable analgesic options, but as mechanism-matched strategies whose value depends on disease stage, dominant pathological driver, and therapeutic window. Overall, precision medicine for PHN should be developed as a stage-dependent decision framework rather than a single biomarker-driven algorithm.

## Conclusion

PHN is a neuroimmune pain disorder driven by VZV reactivation, immune-mediated neuronal injury, oxidative stress, glial activation, and maladaptive peripheral–central sensitization. Current standard therapies remain essential for pain control, but they largely act downstream of the pathogenic cascade and rarely modify disease progression. Emerging mechanism-based strategies, including immunomodulatory, inflammasome-targeted, redox-modulating, gene-based, and RNA-based approaches, may offer opportunities to intervene earlier in the transition from acute neuroinflammation to chronic pain. However, most remain exploratory and require PHN-specific validation of efficacy, safety, delivery, timing, and patient selection. Future management should therefore shift from uniform analgesic escalation toward a stage-specific and phenotype-guided framework integrating cytokine profiles, sensory phenotypes, psychosocial burden, and treatment response, with the goal of preventing chronicity and enabling mechanism-matched disease modification.

## Data Availability

Not applicable.

## Abbreviations

AAV: Adeno-Associated Virus
ATF-3: Activating Transcription Factor-3
CBT: Cognitive Behavioral Therapy
DRG: Dorsal Root Ganglia
DRGS: Dorsal Root Ganglion Stimulation
HSV: Herpes Simplex Virus
HZ: Herpes Zoster
IL: Interleukin
IL-1β: Interleukin-1 Beta
IL-6: Interleukin-6
IL-10: Interleukin-10
IL-18: Interleukin-18
LncRNA: Long Non-Coding RNA
miRNA: MicroRNA
MitoQ: Mitoquinone
NLRP3: Nod-Like Receptor Protein 3
NMDA: N-Methyl-D-Aspartate
NPY: Neuropeptide Y
PHN: Postherpetic Neuralgia
RNAi: RNA Interference
SCS: Spinal Cord Stimulation
shRNA: Short Hairpin RNA
siRNA: Small Interfering RNA
SNRI: Selective Serotonin-Norepinephrine Reuptake Inhibitor
TCA: Tricyclic Antidepressant
TLR4: Toll-Like Receptor 4
TENS: Transcutaneous Electrical Nerve Stimulation
TNF-α: Tumor Necrosis Factor-Alpha
VZV: Varicella-Zoster Virus
